# Pan-Inhibition of Protein Disulfide Isomerase Caused Cell Death through Disrupting Cellular Proteostasis in Pancreatic Ductal Adenocarcinoma Cells

**DOI:** 10.3390/ijms242216467

**Published:** 2023-11-17

**Authors:** Ching-Sheng Hung, Kun-Lin Lee, Wei-Jan Huang, Fang-He Su, Yu-Chih Liang

**Affiliations:** 1 Department of Laboratory Medicine, Wan Fang Hospital, Taipei Medical University, Taipei 11696, Taiwan; oryx@w.tmu.edu.tw; 2 School of Medical Laboratory Science and Biotechnology, College of Medical Science and Technology, Taipei Medical University, Taipei 11031, Taiwan; m609110006@tmu.edu.tw; 3 Ph.D. Program in Medical Biotechnology, College of Medical Science and Technology, Taipei Medical University, Taipei 11031, Taiwan; kunlinleetw@gmail.com; 4 Ph.D. Program in Drug Discovery and Development Industry, College of Pharmacy, Taipei Medical University, Taipei 11031, Taiwan; wjhuang@tmu.edu.tw; 5 School of Pharmacy, College of Pharmacy, Taipei Medical University, Taipei 11031, Taiwan; 6 Traditional Herbal Medicine Research Center, Taipei Medical University Hospital, Taipei 11031, Taiwan

**Keywords:** protein disulfide isomerase, ferroptosis, apoptosis, autophagy, pancreatic ductal adenocarcinoma

## Abstract

The protein disulfide isomerase (PDI) family is a group of thioredoxin endoplasmic reticulum (ER)-resident enzymes and molecular chaperones that play crucial roles in the correct folding of proteins. PDIs are upregulated in multiple cancer types and are considered a novel target for cancer therapy. In this study, we found that a potent pan-PDI inhibitor, E64FC26, significantly decreased the proliferation of pancreatic ductal adenocarcinoma (PDAC) cells. As expected, E64FC26 treatment increased ER stress and the unfolded protein response (UPR), as evidenced by upregulation of glucose-regulated protein, 78-kDa (GRP78), phosphorylated (p)-PKR-like ER kinase (PERK), and p-eukaryotic initiation factor 2α (eIF2α). Persistent ER stress was found to lead to apoptosis, ferroptosis, and autophagy, all of which are dependent on lysosomal functions. First, there was little cleaved caspase-3 in E64FC26-treated cells according to Western blotting, but a higher dose of E64FC26 was needed to induce caspase activity. Then, E64FC26-induced cell death could be reversed by adding the iron chelator, deferoxamine, and the reactive oxygen species scavengers, ferrostatin-1 and N-acetylcysteine. Furthermore, the autophagosome-specific marker, light chain 3B (LC3B)-II, increased, but the autolysosome marker, sequestosome 1 (SQSTM1)/p62, was not degraded in E64FC26-treated cells. Using the FUW mCherry-LC3 plasmid and acridine orange staining, we also discovered a lower number of acidic vesicles, such as autolysosomes and mature lysosomes, in E64FC26-treated cells. Finally, E64FC26 treatment increased the cathepsin L precursor (pre-CTSL) but decreased mature CTSL expression according to Western blotting, indicating a defective lysosome. These results suggested that the PDI inhibitor, E64FC26, might initially impede proper folding of proteins, and then induce ER stress and disrupt proteostasis, subsequently leading to lysosomal defects. Due to defective lysosomes, the extents of apoptosis and ferroptosis were limited, and fusion with autophagosomes was blocked in E64FC26-treated cells. Blockade of autolysosomal formation further led to the autophagic cell death of PDAC cells.

## 1. Introduction

In eukaryotic cells, the endoplasmic reticulum (ER) is responsible for protein folding and post-translational modifications, and it stores calcium ions [1]. As is known, mitochondria are the main organelles that produce reactive oxygen species (ROS) in cells. However, large amounts of ROS are also produced during oxidative protein folding to form disulfide bonds [2,3]. The main enzymes responsible for the formation of disulfide bonds in nascent proteins in the ER are members of the protein disulfide isomerase (PDI) family and the ER oxidoreductase 1 (ERO1) family. They are all thiol oxidoreductases that mainly remain in the ER [2,3]. The active cysteine site in PDIs usually accepts electrons from the free thiol of nascent proteins, so nascent proteins can form disulfide bonds. Afterward, PDI transfers electrons to ERO1, and ERO1 further transfers electrons to O_2_, thereby generating H_2_O_2_. Under normal situations, the H_2_O_2_ can be converted into water and oxygen by many antioxidant enzymes, including superoxide dismutase, catalase, ascorbate peroxidase, glutathione peroxidases, and peroxiredoxin 4, or eliminated by ROS scavengers, such as glutathione and vitamin E [4,5].

PDI family members are ubiquitously expressed in various tissues and cell types in mammals, are especially abundant in secretory tissues, and play vital roles in protein folding and maintaining normal cell functions. Misfolding of proteins can affect their normal biological functions, and the accumulation of misfolded proteins also causes related diseases [6], such as neurodegenerative disorders [7], pulmonary emphysema [8], and cystic fibrosis [9]. In recent years, it was found that PDI family members are overexpressed in various cancers [10], and PDI family members contribute to tumor proliferation and malignancy, and are related to resistance to chemotherapeutic drugs. In most cases, high expression levels of PDI family members have a protective effect on cancer cells and are associated with poor patient survival [11]. In breast cancer stem cells, knockdown of PDI family members, such as *PDIA1*, ER resident protein 44 (*ERp44*), or *ERp57*, inhibits cell proliferation [12], and overexpressions of PDI family members, such as *PDIA3* and *PDIA6*, are associated with invasiveness of primary ductal breast cancer [13]. High expression of anterior gradient protein 2 homolog (*AGR2*), a member of the PDI family, is related to low survival of lung cancer patients [14]. Among PDI family members, PDIA1 is the most critical molecule involved in protein folding and is associated with a variety of diseases, including cancers [15], thrombosis [16], Alzheimer’s disease [17], and diabetes [18]. PDIA1 is overexpressed in a variety of cancers to meet the growing proliferation needs of tumors. Inhibition of PDIA1 by PACMA31 causes toxicity in ovarian cancer cells [19]. Therefore, the development of PDI inhibitors for cancer treatment has become an important topic in recent years [20]. Currently, many PDI inhibitors have been developed; however, those drugs are still in preclinical or early clinical research stages.

Currently, there are no clear molecular action mechanisms describing the ways in which PDI inhibitors cause cell death. In myeloid cancer cells, the PDI inhibitor, CCF642, caused the cleavage of poly(ADP ribose) polymerase (PARP) and caspase-3 and calcium release through apoptosis [21]. However, the PDI inhibitor, LOC14 [22], did not have a significant effect on the cleavage of PARP, and LOC14 even played an anti-apoptosis role in neuron cells [23]. Another study showed that the PDI inhibitor, 35G8, did not cause apoptosis and may have caused cell death through mixed mechanisms of autophagy and ferroptosis [24]. These results indicate that PDI inhibitors may cause cell death through autophagy or ferroptosis, but so far, the action mechanisms of PDI inhibitors remain unclear. This study aimed to understand the molecular mechanisms of the PDI inhibitor, E64FC26, on pancreatic ductal adenocarcinoma (PDAC) cells.

## 2. Results

### 2.1. The PDI Inhibitor, E64FC26, Inhibited Proliferation of PDAC Cells

**Figure 1:**
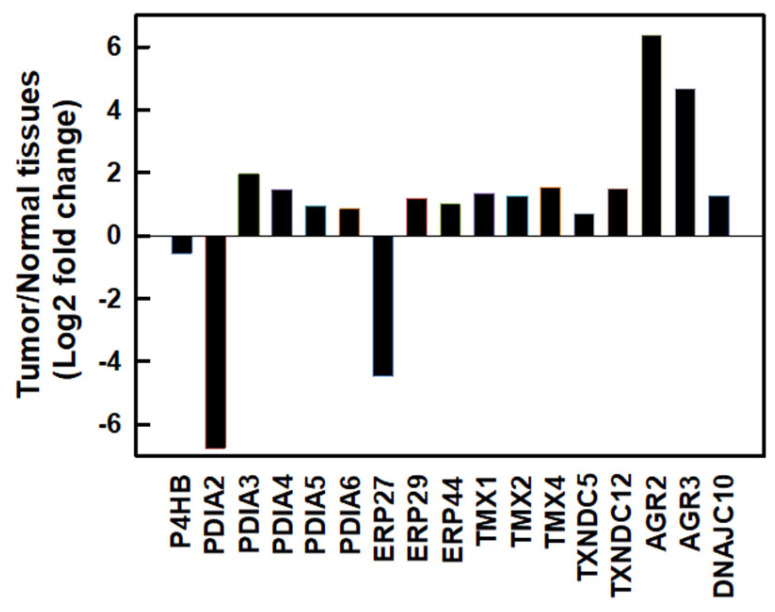
The mRNA expressions of protein disulfide isomerase (PDI) family members in human pancreatic ductal adenocarcinoma (PDAC) tissues. mRNA levels of human PDAC tissues and normal pancreatic tissues were obtained from the GEPIA2 database. PDI family members were selected based on a |log2(fold change)| cutoff of 0.5 and q-value cutoff of 0.01. PDAC tissues, *n* = 179; normal pancreatic tissues, *n* = 171.

We first investigated messenger (m)RNA expressions of PDI family members using the Gene Expression Profiling Interactive Analysis 2 (GEPIA2) database [25] in normal human pancreatic and PDAC tissues. As shown in Figure 1, most PDI family members were upregulated except for *PDIA2* and *ERP27* in human PDAC tissues. Compared to normal pancreatic tissues, the top three members with the highest fold changes in expression in human PDAC tissues were *AGR2* (fold change 6.392), *AGR3* (fold change 4.689), and *PDIA3* (fold change 1.961). These results suggested that PDI family members might play important roles in PDAC cell proliferation.

**Figure 2:**
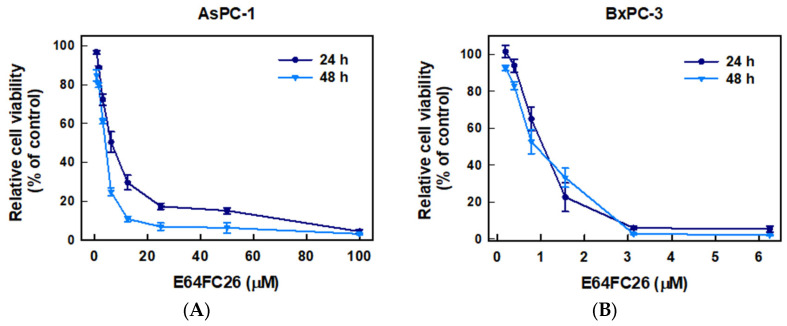
Effects of E64FC26 on the cell viability in human pancreatic ductal adenocarcinoma (PDAC) cells. (**A**) AsPC-1 and (**B**) BxPC-3 cells were treated with different concentrations of E64FC26 for 24 and 48 h, and cell viability was determined by an MTT assay. Each data point is presented as the mean ± S.E. of three independent experiments.

To examine whether inhibition of PDI activity could decrease the proliferation of PDAC cells, we used E64FC26, a novel PDI inhibitor, to treat human pancreatic cancer cell lines AsPC-1 and BxPC-3 cells for 24 and 48 h and determined cell viability by an MTT assay. As shown in Figure 2, E64FC26 significantly decreased the viability of both cell lines in dose-dependent manners. The 50% inhibitory concentrations (IC_50_) with 24 h of treatment were 6.13 ± 0.08 and 0.93 ± 0.33 µM in AsPC-1 and BxPC-3 cells, respectively; and IC_50_ values after 48 h of treatment were 3.41 ± 0.11 and 0.87 ± 0.16 µM, respectively. Prolonged drug treatment for 48 h was more effective than 24 h treatment in AsPC-1 cells, but there was no significant difference in BxPC-3 cells. These results suggest that the E64FC26 PDI inhibitor significantly inhibited proliferation of PDAC cells.

### 2.2. The PDI Inhibitor, E64FC26, Induced ER Stress and the UPR

**Figure 3:**
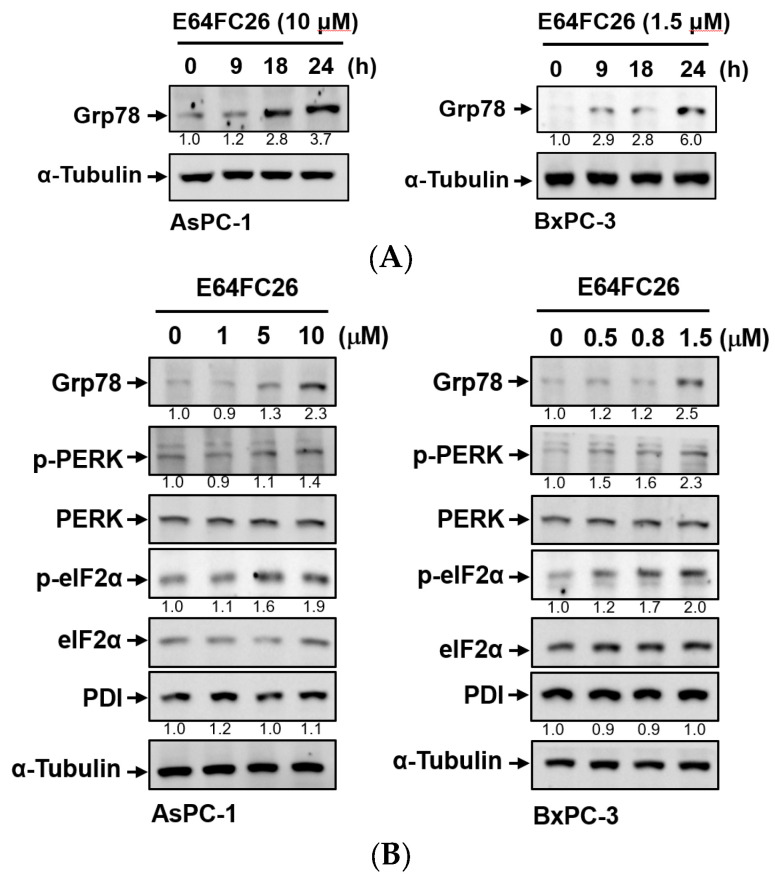
Effects of E64FC26 on endoplasmic reticular (ER) stress- and unfolded protein response (UPR)-related protein expressions in human pancreatic ductal adenocarcinoma (PDAC) cells. (**A**) AsPC-1 and BxPC-3 cells were treated with E64FC26 for different times, and protein levels of ER stress-related proteins were determined by Western blotting. (**B**) AsPC-1 and BxPC-3 cells were treated with different concentrations of E64FC26 for 24 h, and protein levels of ER stress-related proteins were determined by Western blotting. The relative mean intensity of each band (indicated below the bands, *n* ≥ 3) was normalized to the unphosphorylated total protein (including PERK, eIF2α) or α-tubulin loading control.

PDI inhibitors are known to be able to impede the correct disulfide bond formation of nascent proteins and cause protein misfolding [26]. Accumulation of misfolded proteins can increase ER stress and the UPR. To explore whether E64FC26 could induce ER stress and the UPR in PDAC cells, we detected protein expressions of ER stress- and UPR-related signaling pathways. Grp78, a major ER stress-inducible chaperone, was time-dependently upregulated by E64FC26 treatment in both cell lines (Figure 3A). E64FC26 also significantly increased Grp78 expressions in dose-dependent manners in both cell lines (Figure 3B). E64FC26-induced ER stress led to significant PERK phosphorylation, and activated PERK then directly phosphorylated eIF2α in both cell lines. E64FC26 did not change the PDI protein expression in either cell line (Figure 3B). These results suggest that the PDI inhibitor, E64FC26, significantly increased ER stress and the UPR in PDAC cells.

### 2.3. The PDI Inhibitor, E64FC26, Caused Cell Death Partially via Apoptosis

**Figure 4:**
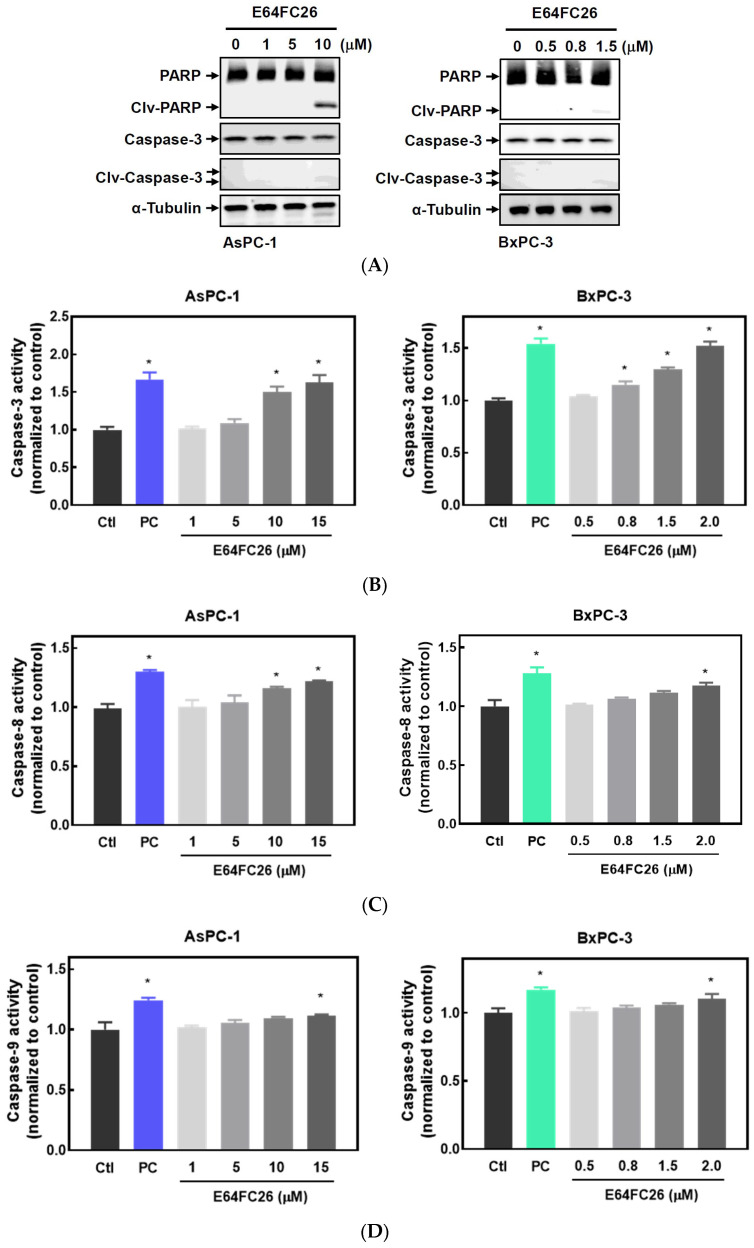
Effects of E64FC26 on activation of caspases in human pancreatic ductal adenocarcinoma (PDAC) cells. (**A**) AsPC-1 and BxPC-3 cells were treated with different concentrations of E64FC26 for 24 h, and protein levels of the poly(ADP ribose) polymerase (PARP) and caspase-3 proteins were determined by Western blotting. (**B**–**D**) AsPC-1 and BxPC-3 cells were treated with different concentrations of E64FC26 for 24 h, and activities of (**B**) caspase-3, (**C**) caspase-8, and (**D**) caspase-9 were detected with a caspase colorimetric assay kit. Concentrations of 1 µM and 100 nM of staurosporine were respectively used as positive controls (PCs) in AsPC-1 and BxPC-3 cells. Each data point is presented as the mean ± S.E. of three independent experiments. * *p* < 0.05 vs. the control (Ctl) group.

It is known that persistent ER stress and the UPR can eventually trigger cell apoptosis [27]. To examine whether the PDI inhibitor, E64FC26, can induce apoptosis in PDAC cells, we detected expressions of cleaved caspases by Western blotting and caspase activities. E64FC26 at 10 and 1.5 µM in AsPC-1 and BxPC-3 cells, respectively, induced very limited expressions of cleaved PARP and cleaved caspase-3 (Figure 4A), but cleaved caspase-8 and caspase-9 were absent from Western blots. To further confirm whether E64FC26 can induce cell apoptosis in PDAC cells, we treated cells with a higher concentration of E64FC26 and directly detected caspase activity with a commercially available caspase activity assay kit. After increasing the E64FC26 concentration to 15 and 2 µM, respectively, in AsPC-1 and BxPC-3 cells, E64FC26 significantly induced caspase-3, -8, and -9 activities using a caspase activity assay kit (Figure 4B). These results suggest that the PDI inhibitor, E64FC26, partially induced cell death through apoptosis in PDAC cells.

### 2.4. PDI Inhibitor E64FC26-Induced Cell Death Might Be Associated with Ferroptosis

**Figure 5:**
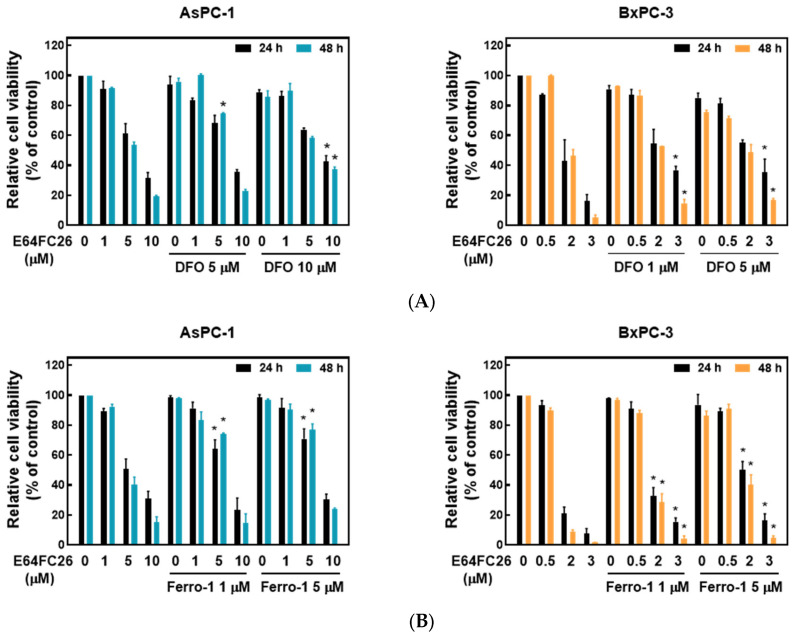

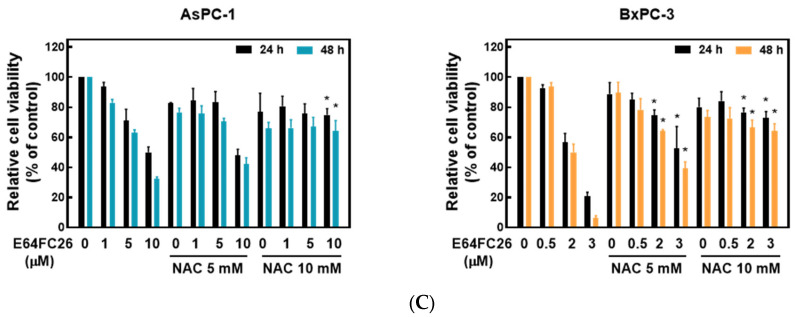
Effects of ferroptosis inhibitors on E64FC26-caused cell death in human pancreatic ductal adenocarcinoma (PDAC) cells. AsPC-1 and BcPC-3 cells were pretreated with (**A**) deferoxamine (DFO) for 6 h, (**B**) ferrostatin-1 (Ferro-1) for 1 h, or (**C**) N-acetylcysteine (NAC) for 24 h, and then treated with E64FC26 for 24 or 48 h. Cell viability was determined by an MTT assay. Each data point is presented as the mean ± S.E. of three independent experiments. * *p* < 0.05 vs. the E64FC26 alone group.

Ferroptosis is a kind of non-apoptotic cell death that depends on iron and lipid peroxidation (LPO) [28]. It was reported that upregulation of ER stress contributes to ferroptosis [29]. To investigate whether E64FC26 caused cell death through ferroptosis, we used the iron-chelating agent, deferoxamine (DFO), and the ROS scavengers, ferrostatin-1 and NAC, to reverse E64FC26-caused cell death. As shown in Figure 5A, pretreatment with 5 and 10 µM of DFO significantly reversed 5 and 10 µM of E64FC26-induced cell death, respectively, in AsPC-1 cells with both 24 and 48 h of treatment. Although DFO alone caused partial cell death, 1 and 5 µM of DFO also reversed 3 µM E64FC26-induced cell death in BxPC-3 cells at both 24 and 48 h of treatment. At both 24 and 48 h, 1 and 5 µM of ferrostatin-1 reversed the 5 µM E64FC26-induced death of AsPC-1 cells, and 2 and 3 µM of E64FC26-induced cell death in BxPC-3 cells (Figure 5B). In addition, 10 mM of NAC prevented cell death under 10 µM E64FC26 treatment for both 24 and 48 h in AsPC-1 cells, and 5 and 10 mM of NAC increased cell survival under 2 and 3 µM of E64FC26 treatment at both 24 and 48 h in BxPC-3 cells (Figure 5C). Interestingly, we detected no significant increase in ROS by the cytosolic ROS sensor, 2’-7’-dichlorodihydrofluorescein diacetate (DCF-DA), in E64FC26-treated cells. These results suggest that E64FC26-caused cell death might be partially mediated through ferroptosis in PDAC cells.

### 2.5. The PDI Inhibitor, E64FC26, Caused Autophagic Cell Death by Blocking Autolysosome Formation

**Figure 6:**
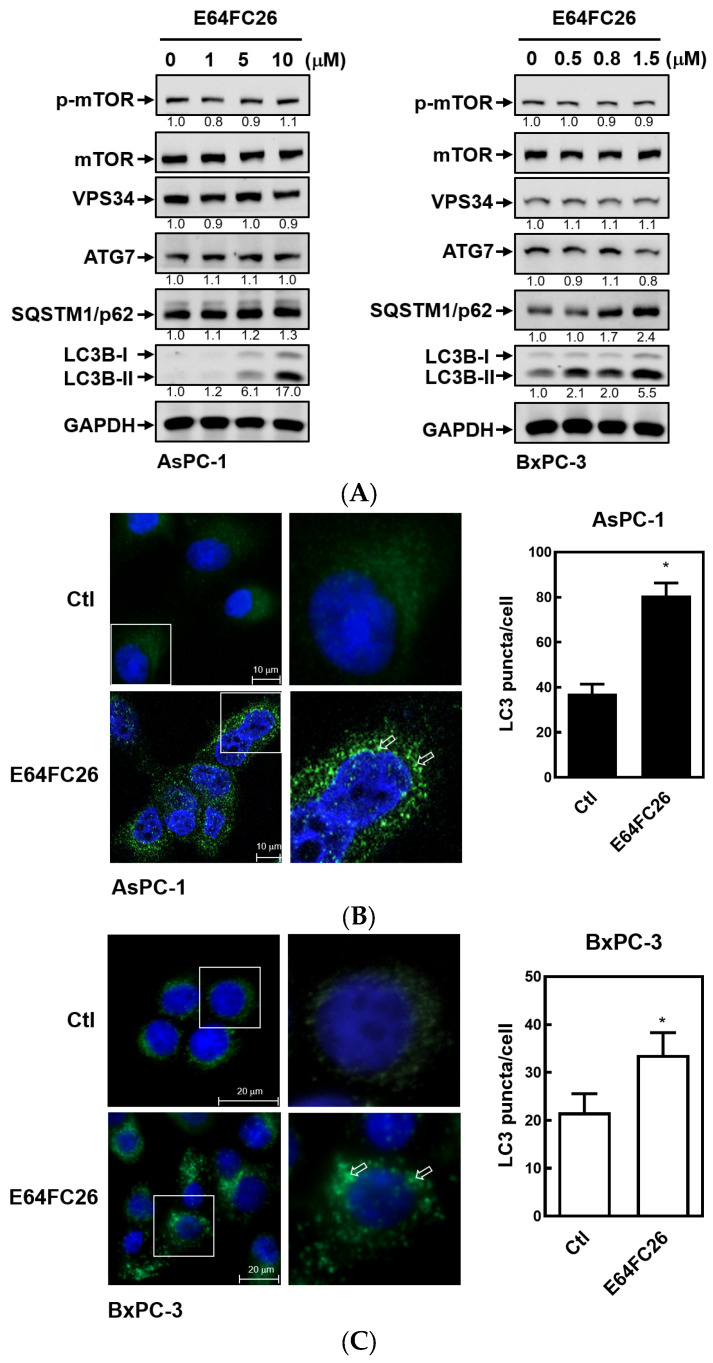
Effects of E64FC26 on autophagy marker protein expressions in human pancreatic ductal adenocarcinoma (PDAC) cells. (**A**) AsPC-1 and BxPC-3 cells were treated with different concentrations of E64FC26 for 24 h, and protein levels of autophagy-related proteins were determined by Western blotting. The relative mean intensity of each band (indicated below the bands, *n* ≥ 3) was normalized to the unphosphorylated total protein (mTOR) or GAPDH loading control. (**B**) AsPC-1 and (**C**) BxPC-3 cells were respectively treated with 5 and 1 µM of E64FC26 for 24 h, and the light chain 3B (LC3B) puncta were detected by IF staining with the CF^®^488A dye (green) and nucleic acid staining with DAPI (blue). Images on the right column are magnifications of the white boxed areas. Representative LC3B puncta are indicated by white arrows. Quantification of LC3 puncta per cell is presented as the mean ± S.E. of three independent experiments. * *p* < 0.05 vs. the control (Ctl) group.

Previous studies demonstrated that PDAC cells have a high basal level of autophagy, which enables them to continue cell proliferation in vivo and in vitro and enhances resistance to chemotherapy and radiation therapy [30,31]. LC3B-II is commonly used as an indicator of mammalian autophagosome formation. To further examine whether the PDI inhibitor, E64FC26, can induce cell death through autophagy, we detected LC3B-II expression by Western blotting and LC3B puncta formation in PDAC cells. As shown in Figure 6A, E64FC26 treatment resulted in LC3B-II accumulation in a dose-dependent manner in both cell lines. Moreover, E64FC26 increased LC3B puncta formation as revealed by IF staining (Figure 6B,C). Expressions of components of autophagy initiation, including VPS34 and Atg7, did not change upon E64FC26 treatment. The final process of autophagy is the fusion of autophagosomes and lysosomes into autolysosomes, which allows degradation of their contents, including SQSTM1/p62. Interestingly, E64FC26 did not induce SQSTM1/p62 degradation but significantly increased SQSTM1/p62 expressions in a dose-dependent manner in both cell lines (Figure 6A). These results suggest that E64FC26 induced incomplete autophagy by increasing the LC3B-II level but failed to degrade SQSTM1/p62.

**Figure 7:**
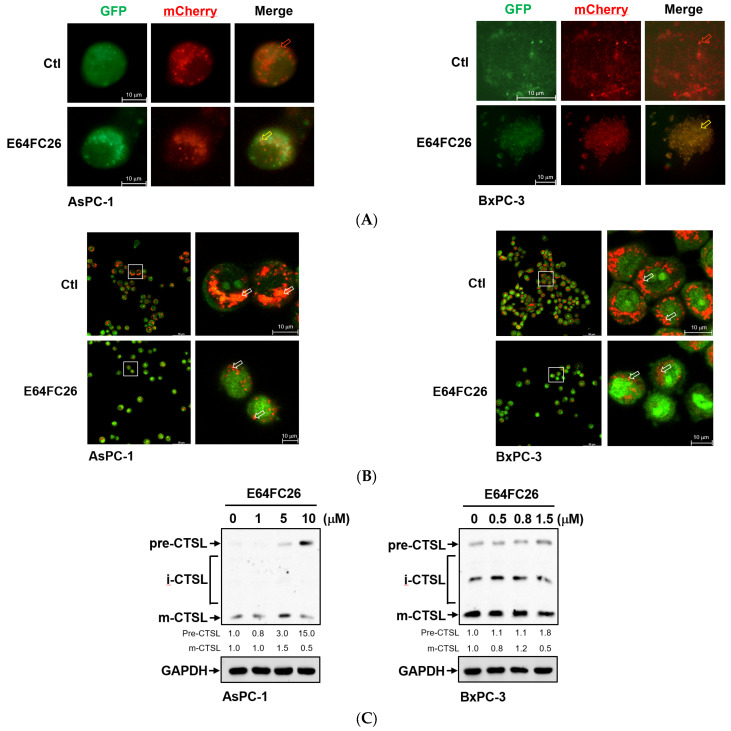
Effects of E64FC26 on autolysosome formation and lysosome functions in human pancreatic ductal adenocarcinoma (PDAC) cells. (**A**) AsPC-1 and BxPC-3 cells were transfected with the FUW mCherry-GFP-LC3 plasmid and then treated with 5 µM and 1 µM E64FC26, respectively, for 24 h. Autophagosomes/autolysosomes were visualized by fluorescence microscopy. Representative autolysosomes and autophagosomes are indicated by red and yellow arrows, respectively. (**B**) AsPC-1 and BxPC-3 cells were treated with 5 µM and 1 µM E64FC26, respectively, for 24 h, and cells were stained with acridine orange for 15 min. Under acridine orange staining, cytoplasmic and nuclear fluorescence was green, and acidic vesicular organelle fluorescence was bright red or orange-red. Images on the right column are magnifications of the white boxed areas. Representative mature lysosomes or acidic autolysosomes are indicated by white arrows. (**C**) AsPC-1 and BxPC-3 cells were treated with different concentrations of E64FC26 for 24 h, and the precursor (pre-), intermediate (i-), and mature (m-) forms of the cathepsin L (CTSL) protein were detected by Western blotting. The relative mean intensity of each band (indicated below the bands, *n* ≥ 3) was normalized to the GAPDH loading control.

To investigate why SQSTM1/p62 was not degraded with E64FC26 treatment, we first transfected the autophagy reporter plasmid FUW mCherry-GFP-LC3 into PDAC cells to express the mCherry-GFP-LC3 fusion protein. The mCherry protein is stable at a neutral pH and in an acidic condition, while the GFP protein is only stable at neutral pH but is acid-labile. Therefore, the mCherry-GFP-LC3 fusion protein displays both green and red fluorescence in the neutral environment of the autophagosome lumen, whereas it only exhibits red fluorescence in the acidic condition of the autolysosome lumen. Compared to control cells, E64FC26 treatment significantly increased yellow fluorescent spots in both cell lines (Figure 7A), indicating that both mCherry and GFP were expressed in autophagosomes of these cells. These results suggest that E64FC26 treatment might block autolysosome formation through inhibiting the fusion of autophagosomes and lysosomes in PDAC cells. To further confirm whether E64FC26 blocks the fusion of autophagosomes and lysosomes, we used AO dye to stain acidic vesicles of cells. As shown in Figure 7B, control cells displayed a lot of reddish-orange fluorescence dots, indicating the formation of mature lysosomes and acidic autolysosomes. However, cells treated with E64FC26 exhibited few reddish-orange fluorescence dots, indicating that E64FC26 blocked autolysosome formation or induced lysosome defects. CTSL is a lysosomal protease that is activated by lysosomal cleavage of pre-CTSL to form mature CTSL. To examine whether E64FC26 impaired lysosome function, we detected expressions of pre-CTSL and mature CTSL by Western blotting in PDAC cells. As shown in Figure 7C, E64FC26 treatment dose-dependently caused the accumulation of pre-CTSL, but decreased mature CTSL in both cell lines. These results suggest that E64FC26 might block autolysosome formation through inducing lysosome defects in PDAC cells.

## 3. Discussion

At least 21 members of the human PDI family have been discovered. The main functions of PDI members include assisting the formation of disulfide bonds in nascent proteins and serving as chaperones to assist the correct folding of proteins. Actively proliferating tumor cells require correct protein folding, and many PDI members are therefore often highly expressed in tumor cells. The development of PDI inhibitors may be a new strategy to fight tumors [20], although no PDI inhibitors have yet entered clinical trials or become Food and Drug Administration (FDA)-approved drugs. Exploring the underlying molecular mechanisms of PDI inhibitors will improve the development of PDI drugs and the feasibility of combining them with other drug treatments. Several PDI members, including *PDIA3*, *PDIA4*, *PDIA6*, *ERP29*, and *TXNDC5*, were found to be highly expressed in numerous cancer types [32]. In addition to those PDI members, *PDIA5*, *ERP44*, *TMX1*, *TMX2*, *TMX4*, *TXNDC12*, *AGR2*, *AGR3*, and *DNAJC10* were also determined to be upregulated in PDAC (Figure 1). It is worth noting that *PDIA1*, *PDIA2*, and *ERP27* are highly expressed in the normal pancreas but are significantly downregulated in PDAC. In this study, we focused on the recently developed novel PDI inhibitor, E64FC26, for treating refractory pancreatic cancer. We found that E64FC26 might disrupt proteostasis and lead to cell death through multiple pathways in PDAC cells. E64FC26 might be developed as a potent anti-PDAC drug either alone or in combination with other anticancer drugs.

Previous studies have found that several reversible inhibitors bind to PDI and inhibits their activities, including 35G8 [24], bepristat 2a [15], ML359 [33], LOC14 [34], and rutin [35]. On the other hand, many covalent inhibitors of PDI have been discovered in the past few years, such as 16F16 [11], CCF642 [21], PACMA31 [19], KSC-34 [36], and E64FC26. In general, reversible inhibitors of PDI are less effective than covalently bound PDI inhibitors, and there is a trend in the number of drugs using covalent inhibitors to treat cancer patients. However, whether covalent inhibitors of PDI can specifically bind to PDI but not other molecules containing SH groups is an important question. The catechol moiety of E64FC26 [(E)-1-nonylidene-3-(trifluoromethyl)-1H-indene-5,6-diol] can be oxidized intracellularly into the o-quinone form, which is further reacts with thiol-containing molecules, such as cysteine residue of proteins and glutathione, to form covalent bonds [37]. The electrophilic trifluoro group of E64FC26 was designed for attaching the nucleophilic cysteines of the PDI active site, but other trifluoro-containing molecules could not become effective PDI inhibitors [38]. Therefore, E64FC26 is considered a PDI inhibitor with higher specificity. E64FC26 was reported to be a potent pan-PDI inhibitor, and is more effective against PDIA1, PDIA3, PDIA4, PDIA6, and TXNDC5 [22]. However, the characteristic of E64FC26 reacting with thiol groups cannot rule out its reaction with non-PDI molecules, especially when body fluids contain very high concentrations of glutathione.

Activating mutations in oncogenic KRAS are very common in pancreatic cancer, where they are persistently activated and induce multiple proliferative signaling pathways. KRAS mutations were found in AsPC-1 cells, while BxPC-3 cells were identified as wild type [39]. Adhesion ability is important for cell migration and affects cell proliferation and invasion in vivo and in vitro [40]. The adherent potential and metastatic activity of BxPC-3 cells were lower than those of AsPC-1 cells [39]. Compared with AsPC-1 cells, lower adhesion and metastasis abilities as well as wild-type KRAS might cause BxPC-3 cells to be more toxic to E64FC26 treatment. As previous studies have demonstrated, gene mutation, such as *KRAS*, EMT phenotype, and cell–cell adhesion could affect drug sensitivity in PDAC cells [41]. AsPC-1 is generally regarded as a drug-resistant cell, but BxPC-3 is a drug-sensitive cell [42]. However, the possibility that the two cells express different levels of PDIs and lead to different toxicities of E64FC26 treatment cannot be ruled out.

Ferroptosis is non-apoptotic cell death associated with iron accumulation and LPO. Many studies have demonstrated that ER stress and ferroptosis can regulate each other [43,44]. Notably, ER stress was found to contribute to ferroptosis. Kuang et al. [45] indicated that palmitic acid-induced ferroptosis is mediated through ER stress and calcium release in colon cancer cells. Whole cigarette smoke condensates induced ferroptosis in bronchial epithelial cells via ER stress caused by induction of hypoxic conditions [46]. Inhibition of cathepsin activity decreased erastin-induced ferroptosis in PDAC cells [29,47], indicating that ferroptosis is a lysosome-dependent type of autophagic cell death. Our current study demonstrated that E64FC26 upregulated ER stress (Figure 3), and E64FC26-induced cell death could be reversed by the iron chelator, DFO, as well as the ROS scavengers, ferrostatin-1 and NAC (Figure 5). Although we detected no increase in ROS of E64FC26-treated cells by the cytosolic ROS sensor, DCF-DA, previous studies using lipophilic antioxidants confirmed that the main cause of ferroptosis is not ROS but LPO [28,48]. It might be better to detect LPO by a membrane-targeted lipid ROS sensor such as BODIPY-C11 in E64FC26-treated PDAC cells. Therefore, E64FC26 might induce ferroptosis through ER stress, but the severity of ferroptosis could be limited by defective lysosomes induced by E64FC26.

Autophagy plays important physical roles in maintaining cellular homeostasis, and has at least three main subtypes: macroautophagy, microautophagy, and chaperone-mediated autophagy (CMA) [49]. Previous studies also demonstrated that misfolded proteins induce ER stress and then activate the adaptive system of the UPR to refold misfolded proteins. In addition, accumulated misfolded proteins are delivered to lysosomes and proteasomes for degradation [50]. The sorting of misfolded proteins into lysosomes is mediated through macroautophagy and CMA. During macroautophagy, the phagophore elongates to encapsulate the misfolded proteins and produce an autophagosome [51]. The autophagosome then fuses with a lysosome to degrade the misfolded proteins. However, CMA-mediated protein degradation involves the Hsc70 chaperone binding to the KFERQ (Lys-Phe-Glu-Arg-Gln) motif of misfolded proteins [52]. Hsc70 client proteins are then sent to lysosomes for degradation. Therefore, lysosomes are important organelles for degrading misfolded proteins, and autophagy is regarded as one of the pathways for lysosomal-dependent degradation [53]. Disruption of autophagy or lysosome function may lead to a failure of cellular proteostasis. Interestingly, ER stress can induce autophagy, which plays a crucial role in cell survival after ER stress [54]. In this study, Grp78, a major stress-inducible ER chaperone and UPR signaling molecule, phosphorylated both PERK and eIF2α, which were significantly upregulated in E64FC26-treated PDAC cells. Results indicated that the PDI inhibitor, E64FC26, might increase the amount of misfolded proteins and then induce ER stress. The induction of macroautophagy and CMA by E64FC26 might result indirectly from ER stress and directly from misfolded proteins. However, neither macroautophagy nor CMA could be completed because of defective lysosomes caused by E64FC26.

The most abundant lysosomal proteases are cathepsins, including serine proteases, cysteine proteases, and aspartyl proteases [55]. Of the 15 classes of cathepsins in humans, 11 classes belong to cysteine proteases, including CTSL. Cathepsins are activated by different proteases and mature in lysosomes. Cysteine cathepsins have intramolecular disulfide bonds to maintain their correct conformation and allow substrates binding along the active-site cleft. Activated cysteine cathepsins depend on their cysteine as the nucleophilic amino acid at active sites [55,56]. In this study, E64FC26 was found to increase pre-CTSL expression but decrease mature CTSL expression in PDAC cells, indicating that E64FC26 might interfere with lysosomal enzyme activities and disrupt proteostasis. It is possible that the PDI inhibitor, E64FC26, decreases activities of cysteine cathepsins through disrupting the normal status of thiol redox or blocking disulfide bond formation of cysteine cathepsins. Lysosomal enzymes are synthesized in ER and delivered in vesicles to the lysosome by the Golgi network. Misfolded proteins caused by E64FC26 might be retained in the ER awaiting proper folding, and the UPR induced by E64FC26 might prompt cells to produce more ER chaperones to restore proper protein folding and processing. In E64FC26-treated cells, enzyme delivery to lysosomes might be reduced, resulting in a decrease in the number or function of lysosomes; on the other hand, the cells might increase the size of the ER. Indeed, fewer acidic vesicles were found in E64FC26-treated cells (Figure 7B). However, we did not examine the activity and number of lysosomes and the size of the ER. More experiments need to be performed in the future to reveal the potential molecular mechanism by which E64FC26 causes lysosome defect.

## 4. Materials and Methods

### 4.1. Chemicals and Antibodies

Deferoxamine (DFO) and E64FC26 were purchased from MedChemExpress (Monmouth Junction, NJ, USA). Ferrostatin-1 was purchased from Cayman Chemical (Ann Arbor, MI, USA), and N-acetylcysteine (NAC) was purchased from Sigma-Aldrich (St. Louis, MO, USA). Primary rabbit polyclonal anti-PKR-like ER kinase (PERK), rabbit polyclonal anti-eukaryotic initiation factor 2α (eIF2-α), and mouse polyclonal anti-cathepsin L (CTSL) were purchased from Santa Cruz Biotechnology (Santa Cruz, CA, USA); rabbit polyclonal anti-phosphorylated (p)-PERK, rabbit polyclonal anti-p-eIF2-α, rabbit polyclonal anti-autophagy related 7 (Atg7), rabbit polyclonal anti-light chain 3 (LC3), rabbit polyclonal anti-cleavage caspase-3, rabbit polyclonal anti-p-mammalian target of rapamycin (mTOR), and rabbit polyclonal anti-mTOR were obtained from Cell Signaling Technology (Danvers, MA, USA); rabbit polyclonal anti-glucose-regulated protein 78 (GRP78), mouse polyclonal anti-α-tubulin, rabbit polyclonal anti-vacuolar sorting protein 34 (VPS34), mouse polyclonal anti-sequestosome 1 (SQSTM1)/p62, and rabbit polyclonal anti-GAPDH were purchased from Genetex (Irvine, CA, USA); and rabbit polyclonal anti-PDI was obtained from Abcam (Waltham, MA, USA).

### 4.2. Cell Culture

The human AsPC-1 and BxPC-3 PDAC cell lines were kindly provided by Prof. Shiow-Lin Pan (Graduate Institute of Cancer Biology and Drug Discovery, Taipei Medical University, Taipei, Taiwan). Both AsPC-1 and BxPC-3 cells were cultured in Roswell Park Memorial Institute (RPMI) medium (Gibco; ThermoFisher Scientific, Waltham, MA, USA) with 10% fetal bovine serum (FBS) and a 1% penicillin/streptomycin solution, and maintained in a humidified incubator at 37 °C with 5% CO_2_.

### 4.3. 3-(4,5-Dimethylthiazol-2-yl)-2,5-diphenyltetrazolium Bromide (MTT) Assay

AsPC-1 and BxPC-3 cells were seeded in 96-well plates at 6 × 10^3^ and 9 × 10^3^ cells/well, respectively. At the end of each experiment, cells were changed to 50 µL of MTT medium and incubated for another 3~4 h. The MTT medium was removed, and 100 µL of DMSO was added to dissolve the MTT formazan, and finally the absorbance at an optical density (OD) of 570 nm was measured on an enzyme-linked immunosorbent assay (ELISA) plate reader [57].

### 4.4. Western Blot Analysis

Cells were seeded at (8~9) × 10^5^ cells in 6 cm dishes. After treatment, cells were lysed in gold lysis buffer (137 mM of NaCl, 20 mM of Tris at pH 7.9, 10 mM of NaF, 1% Triton X-100, 10% glycerol, 5 mM of EDTA, 1 mM of EGTA, 1 mM of phenylmethylsulfonyl fluoride, 10 µg/mL aprotinin, 10 µg/mL leupeptin, 1 mM of sodium orthovanadate, 1 mM of sodium pyrophosphate, and 100 µM of β-glycerophosphate), and 10~30 µg of total cell lysates was used in sodium dodecylsulfate polyacrylamide gel electrophoresis (SDS-PAGE). Proteins in the gel were then transferred to polyvinylidene difluoride (PVDF) membranes and visualized using enhanced chemiluminescence kits (Amersham, Arlington, IL, USA) in an ImageQuant^TM^ LAS4000 Imager system (GE Healthcare Life-Sciences, Taiwan Branch, Taipei, Taiwan) [58]. Relative band intensities of Western blots were quantified using ImageJ software (version 1.54d; National Institutes of Health, Bethesda, MD, USA).

### 4.5. Caspase Activity Assay

Cells were seeded at 7 × 10^5^ cells in 6 cm dishes for 1 day and treated with E64FC26 for another 24 h. Cells were lysed in cell lysis buffer, and 100 µg of total cell lysates was transferred into a 96-well plate. The reaction buffer and individual caspase substrates were added and incubated for 1 h at 37 °C, and then the absorbance at OD 405 nm was measured with an ELISA plate reader according the manufacturer’s instructions (Colorimetric Caspase Assay Kit; BioVision, Waltham, MA, USA) [59].

### 4.6. Transient Transfection

Cells were plated at 4 × 10^5^ cells in 3.5 cm dishes for 1 day and then transfected with the FUW mCherry-GFP-LC3 plasmid (Addgene plasmid no. 110060) using the Lipofectamine^TM^ 3000 reagent (Life Technologies, Taiwan Brand, Taipei, Taiwan). After cells were treated with drugs, green fluorescent protein (GFP) and mCherry fluorescence levels were observed and photographed with a Confocal Spectral Microscope Imaging System (Leica TCS SP5, Singapore) [59].

### 4.7. Acridine Orange (AO) Staining

AsPC-1 and BxPC-3 cells (4 × 10^5^ cells) were cultured in 3.5 cm confocal dishes. Cells were treated with drugs and stained with 2 µg/mL of an AO (3,6-bis (dimethylamino) acridine hydrochloride) solution for 15 min in a cell culture incubator [60]. Fluorescence microscopic images were obtained with a Leica TCS SP5 confocal microscope (Leica Microsystems) using excitation at 458 nm and an emission filter at 480~560 or 590~660 nm.

### 4.8. Immunofluorescence (IF) Staining

A cover slide was plated in a 12-well plate, and 1 × 10^5^ cells were seeded for 1 day. After drug treatment, cells were subjected to IF staining. First, cells were fixed with 4% paraformaldehyde for 20 min, and permeabilized in a 0.5% Triton X-100 solution for 15 min. Then, cells were incubated in a blocking buffer (5% bovine serum albumin in phosphate-buffered saline) for 1 h, in a primary antibody solution (prepared in blocking buffer) overnight at 4 °C, and finally incubated with a CF^®^488A-conjugated secondary antibody (Biotium, Fremont, CA, USA) for 1 h at room temperature. After IF staining, cell nuclei were stained with 1 mg/mL DAPI for 10 min, and cover slides with cells were mounted with 10 µL Fluromount-G (SouthernBiotech, Birmingham, AL, USA). Fluorescence microscopic images were obtained with a Leica TCS SP5 confocal microscope (Leica Microsystems) [59].

### 4.9. Statistical Analysis

Data are presented as the mean ± standard error (S.E.) for the indicated number of independently performed experiments. Statistical analyses were performed using a one-way Student’s *t*-test by GraphPad Prism 9 software, and differences were considered significant at *p* < 0.05.

## 5. Conclusions

**Figure 8:**
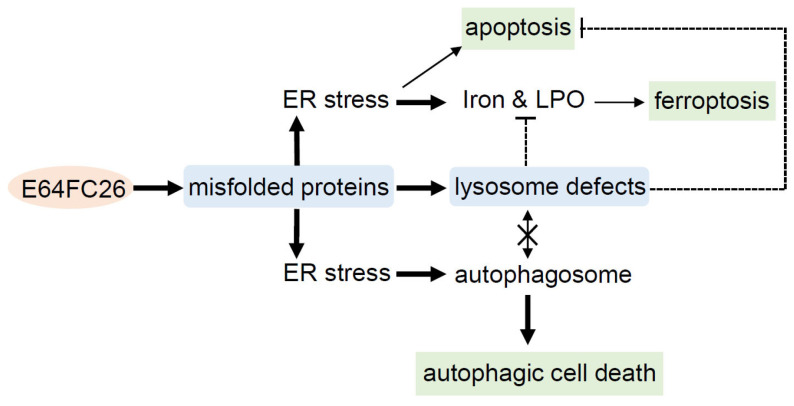
Possible mechanisms of E64FC26-induced cell death in pancreatic ductal adenocarcinoma (PDAC) cells. E64FC26 first induced endoplasmic reticular (ER) stress through impeding proper folding of proteins, and then subsequently caused lysosome defects. ER stress might have initiated ferroptosis and autophagy signals, and cells may have undergone partial apoptosis due to persistent ER stress from higher E64FC26 concentrations. However, defective lysosomes were unable to assist in the apoptosis and ferroptosis processes and hindered fusion with autophagosomes to form autolysosomes. Finally, E64FC26 only induced limited apoptosis and ferroptosis and mainly caused autophagic cell death. Solid lines are used to indicate activated pathways, and dashed lines are used to indicate inhibited pathways. LPO, lipid peroxidation.

This study demonstrated that the PDI inhibitor, E64FC26, might induce ER stress and disrupt proteostasis through hindering the proper folding of proteins, and subsequently lead to lysosome defects. Defective lysosomes restricted the extent of apoptosis and ferroptosis induced by E64FC26-caused ER stress. Furthermore, defective lysosomes failed to form autolysosomes with autophagosomes, ultimately causing autophagic cell death of PDAC cells (Figure 8).

## Data Availability

The data are contained within the article.
